# Clinical Manifestations and Diagnostic Considerations of C7-T1 Single-Level Myelopathy: A Case Series

**DOI:** 10.7759/cureus.60306

**Published:** 2024-05-14

**Authors:** Kento Okamoto, Ryota Kimura, Yuji Kasukawa, Michio Hongo, Daisuke Kudo, Hayato Kinoshita, Yuichi Ono, Naohisa Miyakoshi

**Affiliations:** 1 Orthopaedic Surgery, Akita University Graduate School of Medicine, Akita, JPN; 2 Physical Therapy, Akita University Graduate School of Medicine, Akita, JPN

**Keywords:** clinical manifestation, diagnosis, gait disturbance, cervicothoracic junction, myelopathy

## Abstract

Cervical myelopathy is caused by compression of the cervical spinal cord for any reason. Cervical myelopathy most commonly affects the C5-6 level. However, C7-T1 single-level myelopathy is rare, and neurological findings may be atypical, making diagnosis difficult. We report three cases and discuss their clinical manifestations. Unlike other levels of cervical myelopathy, C7-T1 single-level myelopathy may present with gait disturbance without neurological deficits in the upper extremities. In addition, all three of our cases had different levels of spinal cord compression and locations of sensory deficits; at the C7-T1 level, the spinal cord compression may not correspond to the site of the sensory deficit. These features may help clinicians in the diagnosing of myelopathy.

## Introduction

Cervical myelopathy is a condition that results in chronic progressive compression of the cervical spinal cord due to degenerative disc disease, spondylosis, or other degenerative diseases. Cervical myelopathy often presents with neurological findings including clumsy and/or weak hands and difficulty in fine motor movements, gait disturbance, weakness in the upper extremities, and frequent or urgent urination [1]. Physicians often consider cervical myelopathy to be the primary differential diagnosis that should be prioritized when a patient presents with upper extremity symptoms, especially hand weakness. The C5-6 level is most commonly affected by cervical myelopathy [2]. However, there are few reports on C7-T1 single-level myelopathy [3-7]. Therefore, its clinical features are unelucidated, making diagnosis difficult. Here, we report three cases of C7-T1 single-level myelopathy and discuss their clinical manifestation. This case report highlights important information for the differentiation of the rare C7-T1 single-level myelopathy without overlooking it.

## Case presentation

Case 1

**Figure 1:**
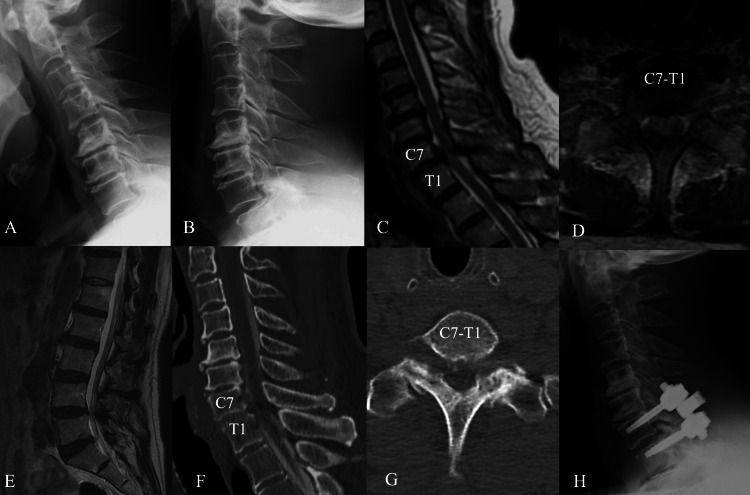
Imaging findings in Case 1 (A) Preoperative cervical spine radiograph in flexion lateral view. C7-T1 level has mild listhesis. (B) Preoperative cervical spine radiograph in extension lateral view. (C) T2-weighted sagittal magnetic resonance imaging (MRI). The spinal cord at the C7-T1 level is compressed and swollen, with mild hyperintensity. (D) T2-weighted axial MRI showing posterior compression of the spinal cord with mild listhesis of C7. (E) T2-weighted lumbar spine MRI showing mild lumbar spinal canal stenosis at L4/5. (F) Sagittal computed tomographic myelography (CTM) showing anterior-posterior compression of the spinal cord. (G) Axial CTM showing contrast thinning at the C7-T1 level and arthrosis of the C7 and T1 facet joints. (H) Lateral view of the postoperative radiograph. C7-T1 laminectomy and posterior instrumented fusion were performed.

A 67-year-old man noticed that he was walking unsteadily for 3 months. His gait was unsteady, and he could not walk without using a cane. He had paresthesia in the anterior thighs and feet. Manual muscle testing (MMT) revealed no muscle weakness in the upper and lower extremities. Patellar tendon reflex (PTR) and Achilles tendon reflex (ATR) were accelerated bilaterally. Radiographs showed no instability in flexion or extension of the cervical spine, but mild listhesis at the C7-T1 level. Magnetic resonance imaging (MRI) in the sagittal plane revealed compression of the spinal cord at the C7-T1 level with mild listhesis and swelling, with a slightly hyperintense area on T2-weighted images. Computed tomographic myelography (CTM) showed compression of the spinal cord in the anteroposterior direction. He was diagnosed with gait disturbance due to C7-T1 myelopathy with listhesis and underwent C7-T1 laminectomy and posterior instrumented fusion (Figure 1). At three years postoperatively, his gait disturbance improved and he was able to walk unassisted.

Case 2

**Figure 2:**
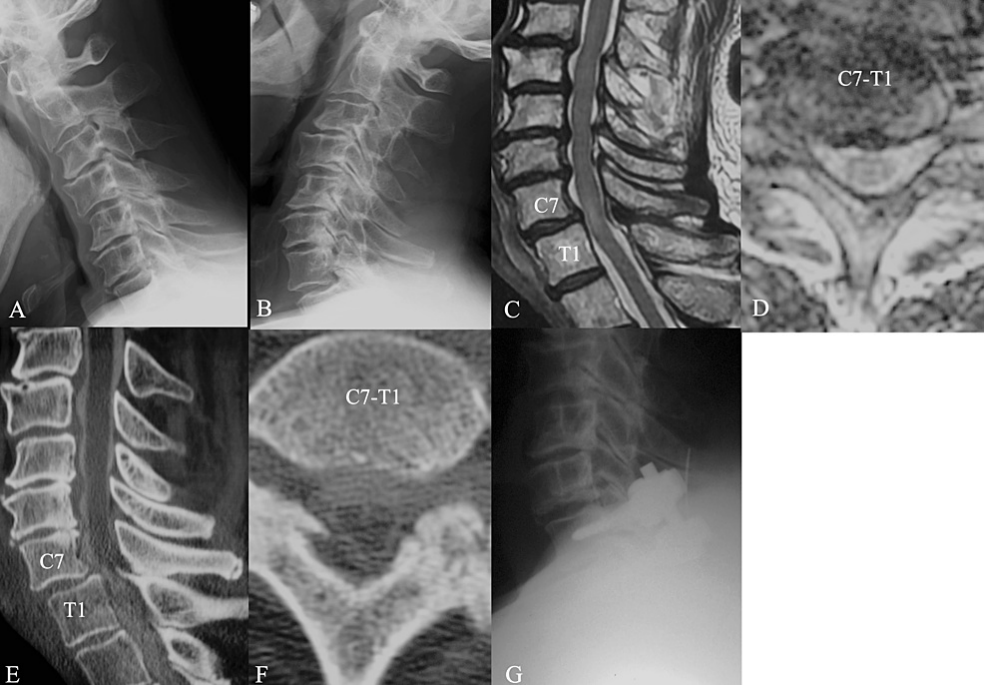
Imaging findings in Case 2 (A) Preoperative cervical spine radiograph in flexion lateral view. C7-T1 level has mild listhesis. (B) Preoperative cervical spine radiograph in extension lateral view. (C) T2-weighted sagittal magnetic resonance imaging (MRI). The spinal cord at the C7-T1 level has high intramedullary intensity. (D) T2-weighted axial MRI at the C7-T1 level, with the spinal cord appearing to be slightly compressed. (E) Paramedian slice of the sagittal computed tomographic myelography (CTM) image showing mild anterior spondylolisthesis at the C7-T1 level and ossification of the ligamentum flavum (OLF) on the right side. (F) Axial CTM view showing OLF compressing the spinal cord. (G) Lateral view of the postoperative radiograph. C7-T1 laminectomy and posterior instrumented fusion were performed.

A 63-year-old man complained of gait disturbance and numbness below his trunk for four months. His gait was spastic. The patient had paresthesia extending from the umbilicus to the lower extremities. MMT ‎revealed no muscle weakness in the upper and lower extremities. Biceps tendon reflex, triceps tendon reflex, PTR, and ATR were all accelerated bilaterally. Radiographs showed no instability in flexion or extension of the cervical spine, but mild listhesis at the C7-T1 level. MRI showed compression of the spinal cord at the C7-T1 level, with high intramedullary intensity on T2-weighted images. CTM showed ossification of the ligamentum flavum (OLF) on the right side with mild listhesis, resulting in a diagnosis of myelopathy at the C7-T1 level. The patient underwent C7-T1 laminectomy and posterior instrumented fusion (Figure 2). At 18 months postoperatively, the preoperative symptoms improved, and the patient was able to walk unassisted.

Case 3

**Figure 3:**
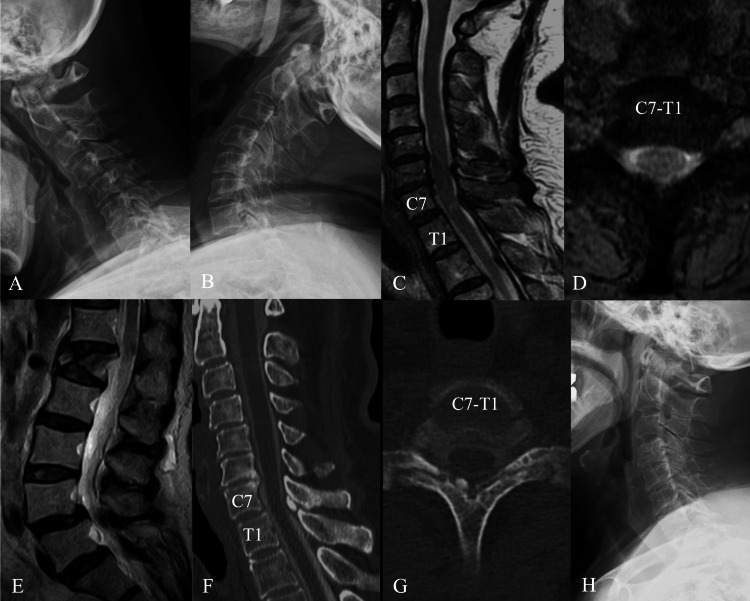
Imaging findings in case 3 (A) Preoperative cervical spine radiograph in flexion lateral view. (B) Preoperative cervical spine radiograph in extension lateral view. There was no instability at the C7-T1 level. (C) T2-weighted sagittal plane magnetic resonance imaging (MRI) showing slightly intramedullary high intensity at the C7-T1 level. (D) T2-weighted axial plane MRI at the C7-T1 level. The spinal cord is compressed posteriorly. (E) T2-weighted sagittal plane MRI of the lumbar spine shows a fracture of the L1 vertebra but no compression of the spinal cord. (F) Paramedian slice of the sagittal computed tomographic myelography (CTM) image showing calcification of the ligamentum flavum on the right side at the C7-T1 level and slight contrast thinning. (G) Axial CTM view showing calcification of the ligamentum flavum. (H) Lateral view of the postoperative radiograph. C7-T1 laminectomy was performed.

A 75-year-old woman was walking unsteadily and would fall frequently six months prior to presentation. Her gait was spastic, which made walking without assistance impossible. The patient had no paresthesia. PTR and ATR were accelerated bilaterally. MMT ‎did not reveal any abnormal results. Radiographs showed no instability in flexion or extension of the cervical spine. MRI of the cervicothoracic junction showed compression of the spinal cord and high intramedullary intensity on T2-weighted images. CTM revealed calcification of the ligamentum flavum on the right side, resulting in a diagnosis of C7-T1 level myelopathy. The patient underwent a C7-T1 laminectomy (Figure 3). The spastic gait improved at the 10-month postoperative follow-up.

## Discussion

The clinical course of these three patients reveals two important issues:

First, C7-T1 single-level myelopathy may present with gait disturbance without upper extremity deficits. Cervical myelopathy typically presents with symptoms such as hand clumsiness and gait disturbance [8]. The uppermost muscle with weakness, uppermost level of sensory disturbance, and decreased or exaggerated tendon reflexes of the upper extremities are used as the analysis index when diagnosing the neurologic level of cervical myelopathy [9]. With regards to muscle weakness, the C8 and T1 myotomes are considered the abductor pollicis brevis and abductor digiti minimi [9]. Hence, a C7-T1 myelopathy would be expected to present with these muscle weaknesses, but none of the three cases presented with them. Cervical myelopathy without upper extremity symptoms has also been reported. Houten et al. reported that 1.2% of patients with cervical myelopathy had no symptoms in the upper extremities [10]. According to our cases as well as published case reports [3-7], 10 of 12 patients do not exhibit any of the aforementioned symptoms, suggesting that upper extremity symptoms are less likely to be features of C7-T1 myelopathy. Since the abductor pollicis brevis and abductor digiti minimi are small muscles, in cases where muscle weakness is experienced, patients may not recognize it as a symptom. Therefore, the patient may not report it to physicians, causing it to be overlooked.

Second, at the cervicothoracic junction, the level of spinal cord compression may not correspond to the site of sensory deficit. Myelopathy often presents with sensory loss and numbness [11]. In cervical myelopathy, the neurological level of diagnosis through sensory deficits has been reported to be the most reliable [9]. If the spinal cord was compressed at the C7-T1 level, sensory deficits would be present from the ring and little fingers to the ulnar aspect of the forearm [12]. However, none of the patients in the three cases developed sensory deficits in the upper extremities. Instead, they had sensory deficits localized from the umbilicus to the lower extremities or lower limbs. Although rare, this discrepancy between cervical cord compression and the level of sensory disturbance has been reported in cervical myelopathy, often referred to as “false localizing signs” [13-15]. Hellmann et al. described cases of cervical cord compression resulting in numbness in areas below the T10 level [14]. Chan et al. reported two cases of sciatica-like leg pain due to cervical cord compression that improved after cervical spine surgery [15]. Our cases suggest that such false localizing signs may occur at a relatively high rate at the cervicothoracic junction.

Most physicians would consider cervical myelopathy as a differential diagnosis in the presence of upper extremity symptoms, hand numbness, and hand clumsiness. However, when a patient presents with only numbness or pain and weakness in the lower extremities, the diagnosis of cervical myelopathy is reportedly delayed owing to suspicions of lumbar disease [16,17]. Thoracic myelopathy is associated with a high rate of lower extremity numbness, subjective lower extremity weakness, and unsteady gait [18]. OLF as well as the posterior longitudinal ligament and intervertebral disc herniation are some conditions that cause thoracic myelopathy. However, OLF, which is relatively common, tends to cause compression of the lower thoracic spinal cord [18]. Since C7-T1 has the smallest range of motion in all directions compared to the other cervical vertebrae, one would expect less arthrosis and disc degeneration in the joints [5,7]. Therefore, physicians may miss upper thoracic findings, such as OLF, calcification of the ligamentum flavum, facet joint arthrosis, and so on. If these symptoms are present and thoracic myelopathy is suspected, the possibility of lesions at C7-T1 should be considered.

## Conclusions

C7-T1 single-level myelopathy may present with gait disturbance without deficits in upper extremity functions, and at the cervicothoracic junction, the level of spinal cord compression may not correspond to the site of sensory deficit. These symptoms and physical findings can complicate the differential diagnosis. For an appropriate diagnosis, physicians must note that myelopathy can often be seen with gait disturbance alone.
